# Tactile Imaging Markers to Characterize Female Pelvic Floor Conditions

**DOI:** 10.4236/ojog.2015.59073

**Published:** 2015-08-26

**Authors:** Heather van Raalte, Vladimir Egorov

**Affiliations:** 1 Princeton Urogynecology, Princeton, NJ, USA; 2 Artann Laboratories, Trenton, NJ, USA

**Keywords:** Biomechanics, Soft Tissue, Tactile Imaging, Prolapse, Tissue Elasticity, Elastography

## Abstract

The Vaginal Tactile Imager (VTI) records pressure patterns from vaginal walls under an applied tissue deformation and during pelvic floor muscle contractions. The objective of this study is to validate tactile imaging and muscle contraction parameters (markers) sensitive to the female pelvic floor conditions. Twenty-two women with normal and prolapse conditions were examined by a vaginal tactile imaging probe. We identified 9 parameters which were sensitive to prolapse conditions (*p* < 0.05 for one-way ANOVA and/or *p* < 0.05 for *t*-test with correlation factor *r* from −0.73 to −0.56). The list of parameters includes pressure, pressure gradient and dynamic pressure response during muscle contraction at identified locations. These parameters may be used for biomechanical characterization of female pelvic floor conditions to support an effective management of pelvic floor prolapse.

## 1. Introduction

Many of pelvic floor disorders, including prolapse, stress urinary incontinence, sexual dysfunction, congenital anomalies and others, are clearly manifested in the mechanical properties of pelvic organs. Therefore, mapping a response to applied pressure or load within the pelvic floor opens new possibilities in biomechanical assessment and monitoring of pelvic floor conditions.

When the human finger palpates soft tissue, the brain tries to estimate the pressure response versus the finger motion. While different tissue characteristics may be detectable with side-by-side palpation, the human finger cannot distinguish even substantial deviations in tissue elasticity for two locations if they are separated in time or space. The brain cannot remember the finer aspects of tissue elasticity reliably. We also rely on visual assessments to augment palpated properties, such as the visually perceived tissue distention with Valsalva or coughing, in order to gather functional information of the vaginal tissue. The tactile imaging device, on the other hand, offers recordable and reproducible measurements for tissue evaluation [1].

Earlier, we designed a prototype of the Vaginal Tactile Imager (VTI) for visualization and assessment of the elastic properties of pelvic floor tissues [1]–[4]. The objective of this study is to identify specific tactile imaging and muscle contraction markers to characterize female pelvic floor conditions.

## 2. Materials and Methods

### 2.1. Tactile Imaging

We define tactile imaging as a medical imaging modality that translates the sense of touch into a digital image. The tactile image is a function of *P*(*x, y, z*), where *P* is the pressure on soft tissue surface under applied deformation and *x, y, z* are coordinates where pressure *P* was measured. Tactile imaging closely mimics manual palpation, since the probe of the device with a pressure sensor array mounted on its face acts similarly to human fingers during clinical examination, slightly deforming soft tissue by the probe and detecting resulting changes in the pressure pattern. The tactile image is a pressure map on which the direction of tissue deformation must be specified. We calculated within the acquired tactile images the spatial gradients ∂*P*(*x, y*)/∂*y* directed to anterior/posterior (*y*-coordinate) from the vaginal channel (*x*-coordinate) at the regions of interest. The solid vaginal probe with pressure sensors along its two opposite sides allows high-resolution recording of dynamic response from pelvic floor muscles during muscle contractions.

### 2.2. Vaginal Tactile Imager

**Figure 1:**
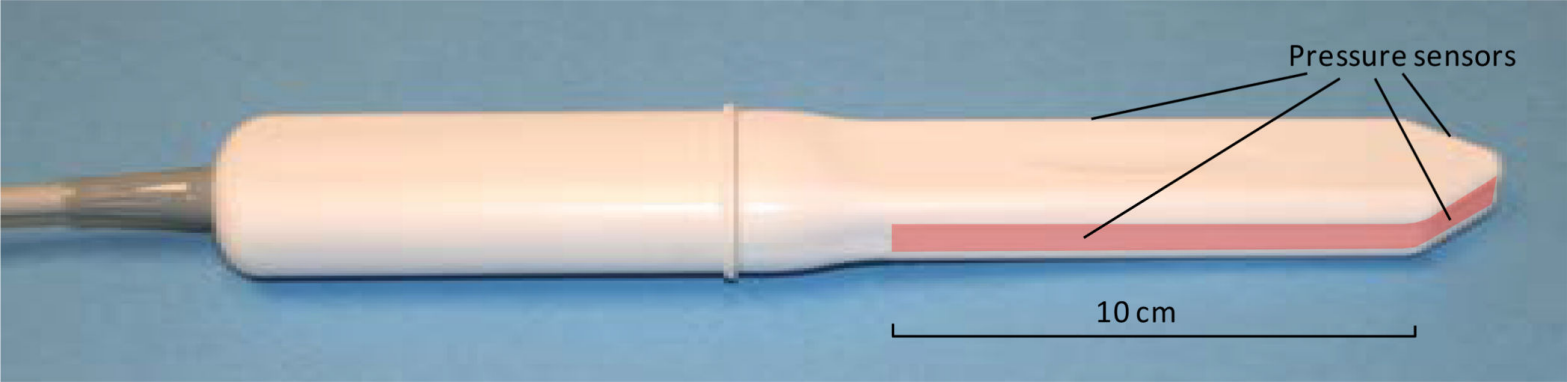
Vaginal probe. Pressure sensors are aligned on the outer surface of the probe (highlighted on the image).

The VTI probe, as shown in Figure 1, is equipped with 96 pressure (tactile) sensors positioning every 2.5 mm along the both sides of the probe, an orientation sensor (accelerometer) and temperature sensors with micro-heaters. During the clinical procedure, the probe is used to acquire pressure responses from the vaginal walls. The VTI examination procedure includes data collection from all of the segments of the vagina. During an examination, data are sampled from the probe sensors and displayed on the VTI monitor in real time. The resulting pressure maps (tactile images) of the vagina integrate all of the acquired pressure and positioning data for each of the pressure sensing elements. In addition, the VTI records the dynamic contraction for pelvic floor muscles. The probe surfaces that contact the vaginal walls are preheated to human body temperature. A lubricating jelly is used for patient comfort and to provide reproducible boundary/contact conditions with deformed tissue; these conditions are classified as slip boundary conditions. The tactile probe measures an applied pressure, but not force. Force is a vector and by definition has amplitude and direction. The pressure sensors designed for VTI probe are not sensitive to tangential component of a force which may arise during probe motion and the sensors measure *Pressure = Force* (*orthogonal component*)*/Area*. This probe can be used not only for tissue compression in orthogonal direction to the tissue surface during the probe insertion (Tests 1), but it can be used for tissue compression during probe elevation (Test 2), for pressure pattern acquisition during probe rotation (Test 3) and pelvic floor muscle contraction (Test 4). The probe maneuvers in Tests 1 – 3 allow accumulation of multiple pressure patterns from the tissue surface to compose an integrated tactile image for the investigated area using a proprietary image composition algorithm like we developed for the breast and prostate [5] [6].

The VTI software includes data analysis tools and reporting functions. It visualizes the anatomy of the vagina incorporating spatial measurements, pressure levels, calculated pressure gradients within the pressure maps, and assesses the pelvic floor muscle contraction capability (muscle strength).

The examination procedure allows 4 tests: 1) probe insertion, 2) elevation, 3) rotation, and 4) voluntary muscle contraction. These tests provide the following information:

Test 1: Tactile image for vaginal anterior and posterior compartments along the entire vagina; pressure gradients and anatomical sizes can be calculated.

Test 2: Tactile image for apical anterior and posterior compartments which related to pelvic floor support structures; pressure gradients and anatomical sizes can be calculated.

Test 3: Tactile images for left and right sides of vagina (circumferential tactile image from vaginal walls); anatomical sizes can be calculated.

Test 4: Dynamic pressure response from voluntary PFM contractions recorded from for the opposite sides along the entire vagina; static and dynamic components can be separated.

The VTI measurement accuracy established with tissue models: ±3 mmHg for pressure, ±0.5 degree for probe orientation and ±0.1°C for measuring the temperature inside the probe on the surface of the micro-heaters. The probe was calibrated before every examination; it was cleaned and disinfected between patients. Because of the angled tip probe design (Figure 1), it is possible to translate the probe’s linear motion during Test 1 into vaginal wall deformation from the center of vaginal channel. Tests 1 – 3 reflect passive tissue measurements (no PFM contraction).

### 2.3. Population Description

Twenty two women were enrolled in an observational study (clinical trials identifier NCT01848626 at http://clinicaltrials.gov) and underwent VTI examination. The analyzed data set included 20 subjects aged from 41 to 70 years. Prior to the VTI examination, a standard physical examination was performed including a bimanual pelvic examination and Pelvic Organ Prolapse Quantification (POP-Q) [7]. The pelvic floor conditions were categorized by prolapse staging based of maximum stage from anterior, posterior and uterine prolapse. Using this approach we found that 4 subjects had normal pelvic floor conditions, 4 with pelvic organ prolapse Stage I, 7 with Stage II, 4 with Stage 3 and 1 with Stage IV. Two subjects were excluded from analyzed data set because they have had a prior pelvic floor surgery. The clinical protocol was approved by the local Institutional Review Board and all women gave written informed consent. The study was done in compliance with the Health Insurance Portability and Accountability Act. The VTI images were obtained and recorded at the time of scheduled routine urogynecologic visits.

Total study workflow comprised of the following steps:

1) Recruiting women who routinely undergo vaginal examination as a part of their diagnostic treatment of concerned areas;
2) Acquisition of clinical diagnostic information related to the studied cases by standard clinical means;
3) Performing a VTI examination in lithotomic position;
4) Analyzing tactile images and assessment of potential markers such pressure, pressure gradients and muscle contracting responses suitable for pelvic floor characterization.

Additionally, the patients were asked to assess pain and comfort level of VTI examination relative to manual palpation.

### 2.4. Statistical Analysis

The tactile imaging data from all the examinations were consolidated into a single dataset. Image reviewers had no knowledge of the subject’s pelvic floor conditions to avoid bias in the data processing. The clinical information (staging, age and parity) was then added to this dataset after the tactile imaging data (pressure, pressure gradients, muscle contracting response) were finalized.

One-way analysis of variance (ANOVA), paired *t*-test (normal plus State 1 *vs* States 2 – 4), and Pearson’s correlation coefficients were calculated to determine whether the various parameters showed dependence on the pelvic floor conditions using MATLAB 6.1 (Math Works, Natick, MA). For visual evaluation of the analyzed clinical data distributions we used the notched boxplots [8] showing a confidence interval for the median value (central horizontal line), 25% and 75% quartiles. The spacing between the different parts of the box helps to compare variance. The boxplot also identifies skewness (asymmetry) and outlier (small cross). The intersection or divergence of confidence intervals for two patient samples is a visual analog of the paired *t*-test.

## 3. Results

All 22 enrolled women were successfully examined with the VTI and tactile images of vagina were recorded and stored. A typical examination consisting of four steps takes 1 to 2 minutes and the acquired data is used to generate a patient examination report.

**Figure 2:**
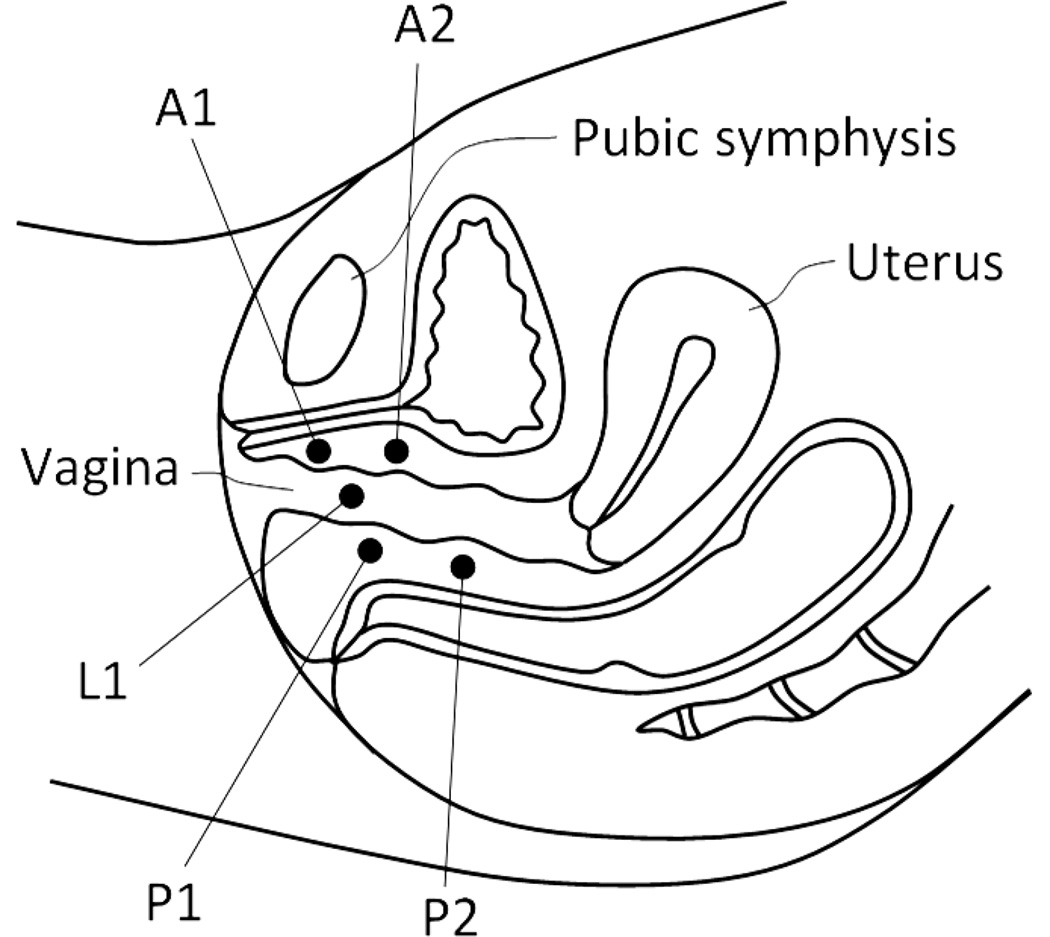
Locations of the VTI markers identified within the pelvic floor. A1 and A2 are within the anterior compartment, P1 and P2 in the posterior compartment and L1 in the lateral compartments (left and right sides).

Upon reviewing the images, several areas were identified with consistently observed pressure peaks across VTI scans. They were selected as the marker sites for analysis. Specifically, the following locations along the pelvic floor were used for marker calculations: A1—anterior in the vicinity of hymen with maximum pressure feedback; A2—the second pressure peak along to anterior toward to proximal part; P1—posterior in the vicinity of hymen with maximum pressure feedback; P2—the second pressure peak along to posterior toward to proximal part; L1—vaginal sides with maximum pressure peaks in the vicinity of hymen. Figure 2 illustrates the listed locations. The pressure peaks used for A1, A2, etc. are not fixed (e.g. 2 cm from the introits), but varied among the patients.

**Figure 3:**
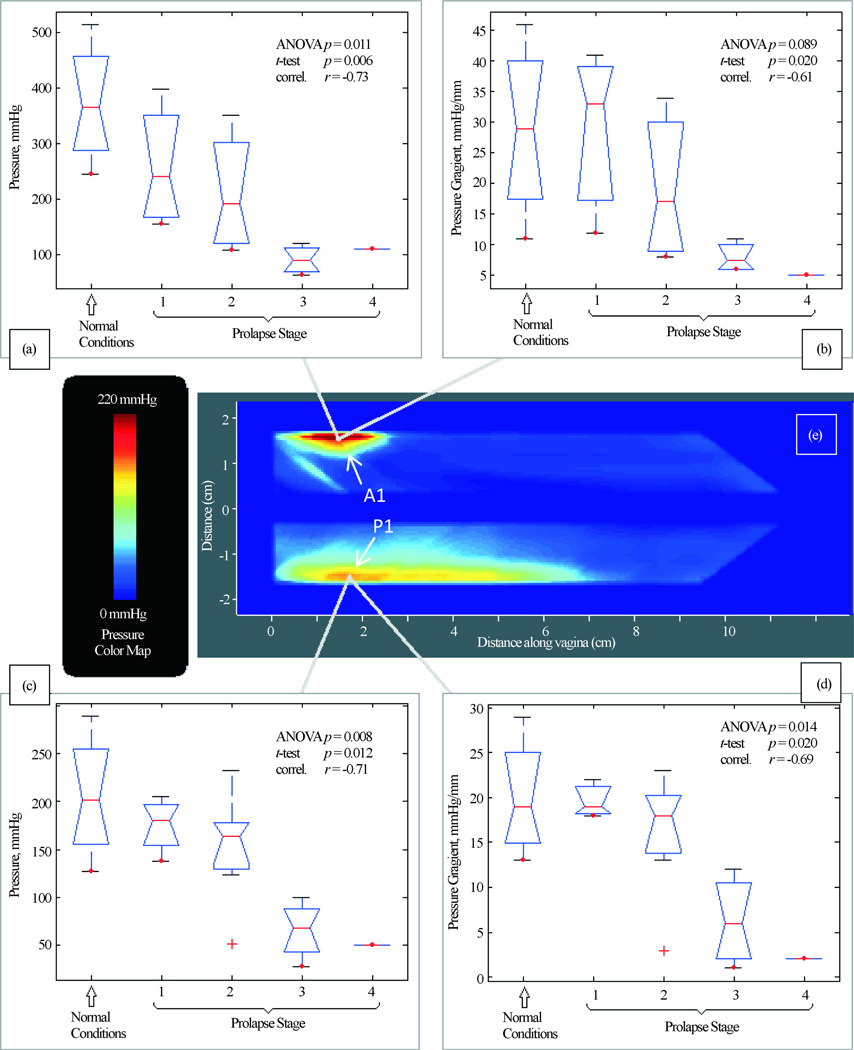
Test 1—probe insertion results. Tactile imaging markers at distal anterior (panels (a) and (b)) and distal posterior (panels (c) and (d)) which are sensitive to varying degrees of prolapse. Panel (e) shows a typical pressure response map (tactile image) for this test.

During Test 1 (Probe Insertion) we have identified four (4) parameters that are potential markers for pelvic floor conditions (see Figure 3). They demonstrate correlation from −0.73 to −0.61 with pelvic floor conditions (normal and stage I-IV). These 4 parameters for A1 and P1 locations show a mild correlation from −0.40 to −0.13 with patient age and a mild correlation from −0.37 to −0.22 with parity. No significant correlations were found for other locations in this test.

**Figure 4:**
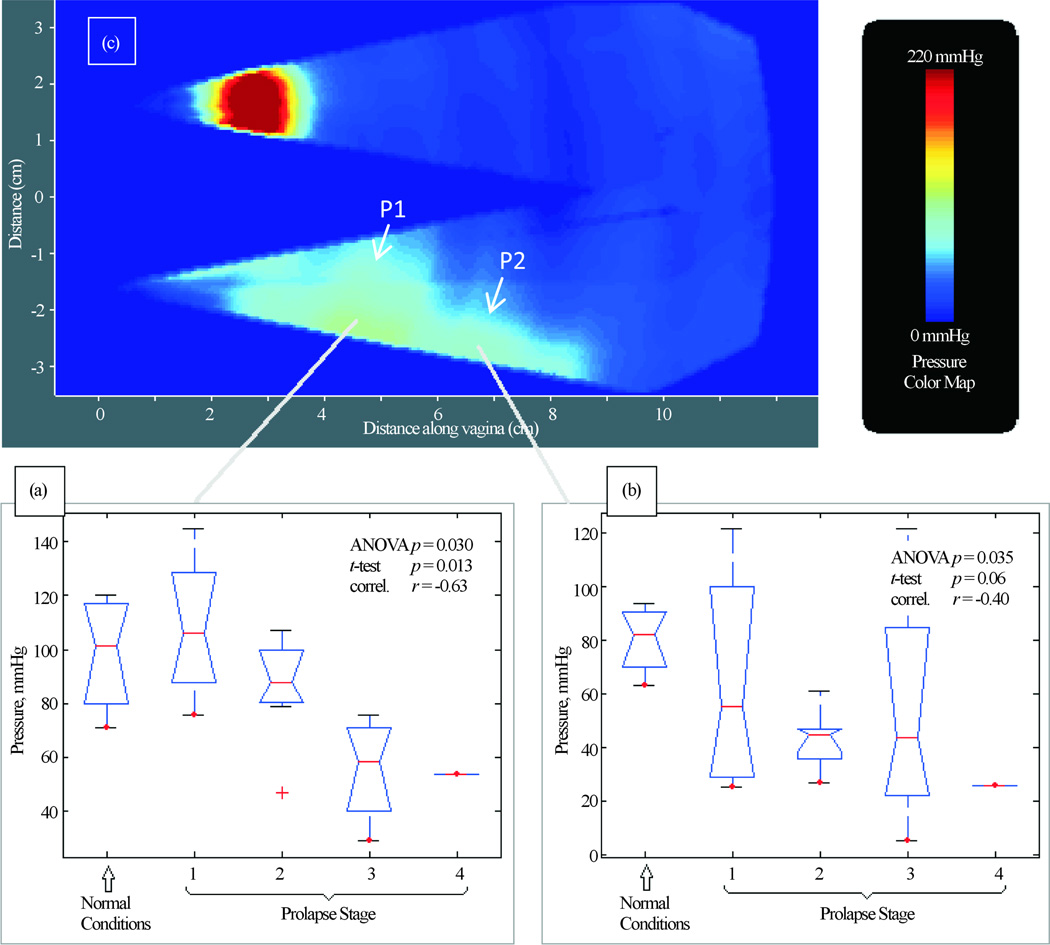
Test 2—probe elevation results. Tactile imaging markers at posterior (panels (a) and (b)) which are sensitive to varying degrees of prolapse. Panel (c) shows a typical pressure response map (tactile image) for this test.

Figure 4 presents our findings for Test 2 (Probe Elevation). We have identified two (2) parameters for P1 and P2 locations that are potential markers for pelvic floor conditions. They demonstrate a correlation from −0.63 to −0.40 with pelvic floor conditions (normal, Stages I-IV). These parameters showed a mild-moderate correlation with patient age (−0.52 and 0.26) and parity (−0.26 and 0.13). No significant correlations were found for other locations in this test.

**Figure 5:**
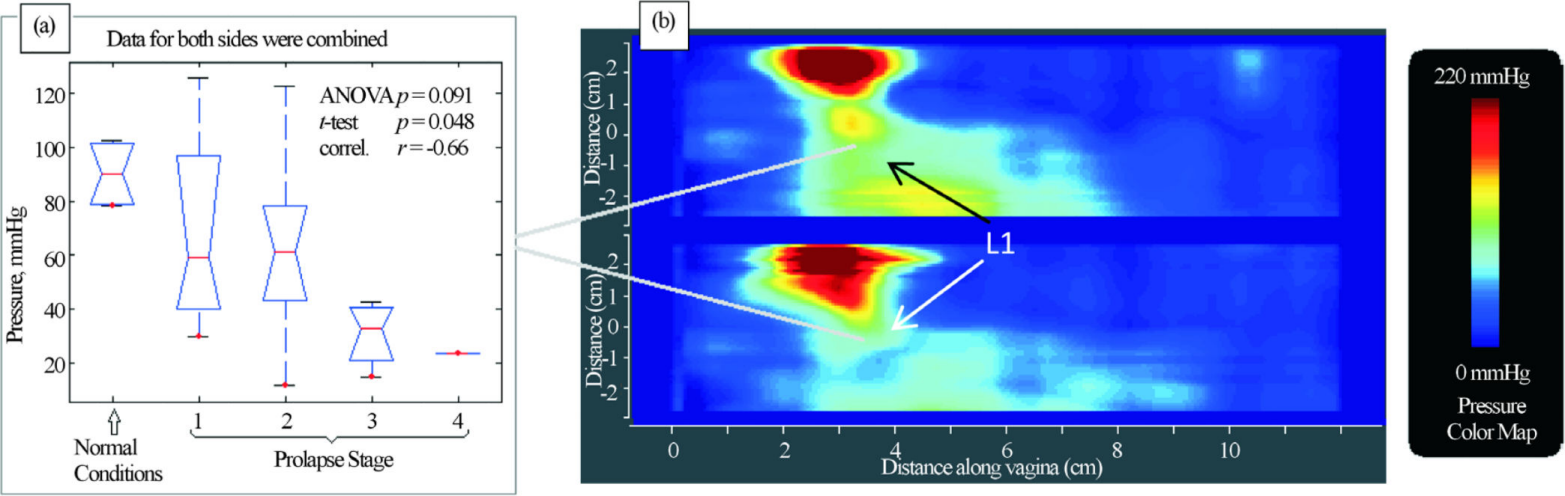
Test 3—probe rotation results. Tactile imaging marker at distal lateral location (panel (b) which is sensitive to varying degrees of prolapse. Panel (a) shows a typical pressure response map (tactile image) for this test.

Figure 5 presents our findings for Test 3 (Probe Rotation). We have identified one (1) parameter that is potential marker for pelvic floor conditions. It demonstrates correlation 0.66 with pelvic floor conditions (normal and stage I-IV) and weak or no correlation with patient age and parity.

**Figure 6:**
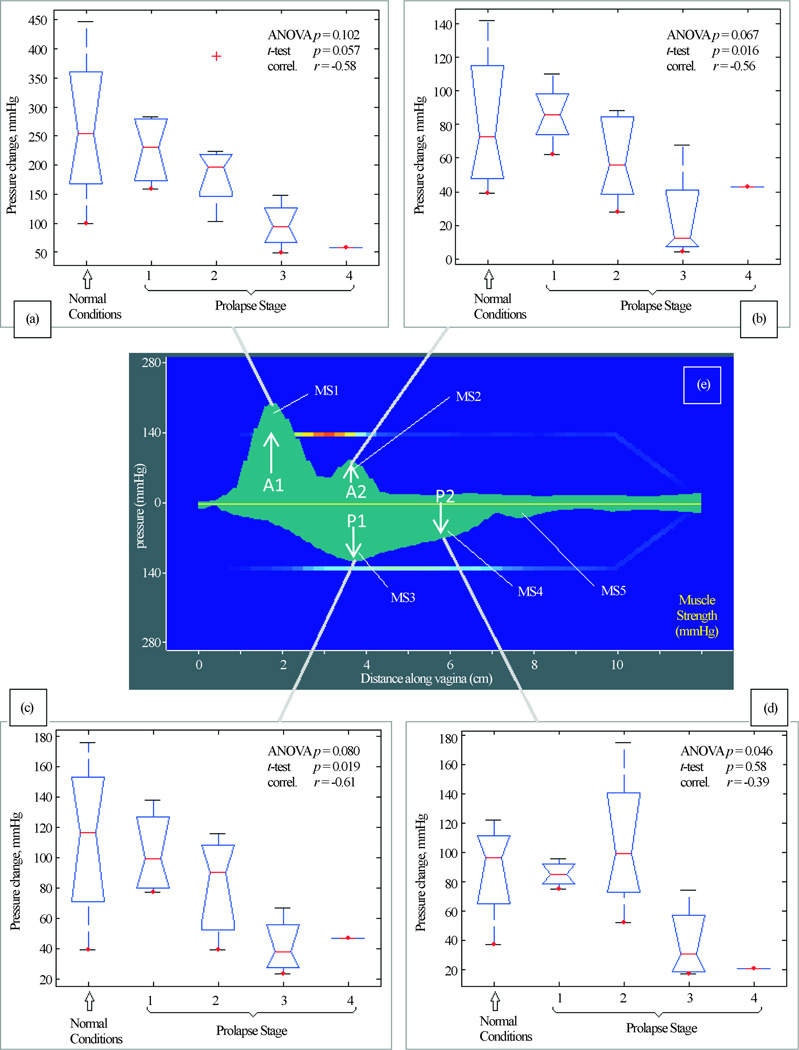
Test 4—voluntary PFM contractions. Muscle contraction capabilities at anterior (panels (a) and (b)) and posterior (panels (c) and (d)) which are sensitive to varying degrees of prolapse. Panel (e) shows distribution of maximum pressure response for anterior and posterior compartments for this test.

Figure 6 presents our findings for Test 4 (Muscle Contraction). We have identified four (4) parameters that are potential markers for pelvic floor conditions. They demonstrate correlation from −0.61 to −0.39 with pelvic floor conditions (normal and stage I-IV). These parameters show weak correlation with patient age and parity.

In Test 4 (Voluntary PFM Contractions) we observed 5 peaks as shown in Figure 6. Four of them (MS1-MS4) were identified as potential markers for pelvic floor characterization, as they demonstrate a correlation with pelvic floor conditions. The peak MS5 demonstrated variability from patient to patient and in this subset of patients, did not show any correlation with degree of prolapse.

Seventy three percents (73%) of the patients classified the VTI pain as none, 24% as mildly painful and 3% as a painful, on a 4-degree scale: none, mildly painful, painful, and severely painful. The patients were asked also to assess comfort level of VTI examination relative to manual palpation: 54% stated the VTI procedure was more comfortable, 36% the same and 10% less comfortable than manual palpation. No adverse events were reported.

## 4. Discussion

We report a new approach to image and measure the behavior of the pelvic floor support system under vaginal tissue deformation and muscle contraction in women with and without prolapse. Using tactile imaging probe as shown in Figure 1, we found that patients with prolapse have pressure gradient measurements decreased 2 – 4 fold (200% – 400%) at specific locations (see Figure 3(b), Figure 3(d)) which can be interpreted as 2 – 4 fold softer than in patients with normal support and pelvic floor muscle contractive capabilities (muscle strengths) decrease up to 5 times (500%) (see Figure 6). These results clearly demonstrate that women with prolapse have significantly mechanical differences within the vaginal and surrounding pelvic floor supportive systems. While this is not new or surprising clinical finding [9] [10], biomechanical values of these changes with prolapse are acquired *in vivo* for the first time (Figure 3–Figure 6) which may add a valuable dimension to our current assessment of pelvic floor disorders.

In the current study, we have identified 11 parameters as potential markers for pelvic floor characterization with VTI use. We found that 9 of 11 parameters show statistically significant differences for prolapse conditions with *p* < 0.05 for one-way ANOVA and/or *p* < 0.05 for *t*-test. These 9 parameters have correlation factor (*r*) from −0.73 to −0.56. These parameters demonstrate a mild-moderate correlation with women age and parity for specified sample size. The results of this study demonstrate that the vaginal tactile images can be acquired and coupled with functional pelvic muscle assessment in one VTI examination.

In addition to recording tactile feedback during the tissue deformation, the VTI obtains measurement of muscle strength and allows evaluation of the relative functional impact of muscle contraction on measured biomechanical properties. The ability to assess and map pelvic floor muscles along entire vagina with the resolution of 2.5 mm is a novel measurement in pelvic floor assessment. The VTI pressure graphs for Test 4 (see Figure 6) are smoothed to 0.5 mm for better visual perception understanding that sharp pressure transitions in the tissue are not possible. To our knowledge, it is the first time the five pressure peaks were observed during pelvic floor muscle squeezing (see Figure 6(e)). One of the sites measured (A1) (see Figure 6(e)), is potentially exaggerated or an artifact because of the pubic bone, but this peak does have a lateral component which contradict sole resistance from the static structure. These peaks have a complex, dynamic pattern and require further investigation. One potential limitation is that the measurements were taken only with the probe in an anterior-posterior plane. Future evaluation with the probe in varied positions may better assess and document asymmetrical findings and provide a circumferential assessment of muscle function.

To fully characterize tissue as a mechanical system a great number of parameters are needed including the shear and Young’s moduli, bulk compressional modulus, nonlinearity, Poisson’s ratio, viscosity, poroelastic parameters, anisotropy and heterogeneity indices, etc. However, in most practical cases, there is no need to have a comprehensive mechanical characterization of the tissue of interest and even just one elasticity parameter, such as Young’s modulus (E), may be sufficient to address diagnostic tasks. Detection of a mechanical heterogeneity by manual palpation is based exclusively on sensing the variations of the Young’s modulus of tissue [11] [12], which may change by hundreds of percents from tissue to tissue and due to pathological or physiological conditions [11]–[15].

Generally, inverse problem solution for 3-D tactile image *P*(*x, y, z*), would allow reconstruction of tissue elasticity distribution (*E*) as function of the same coordinates *E*(*x, y, z*). Unfortunately, the inverse problem solution is hardly possible for most real objects because it is non-linear and ill posed problem. But it seemed out that Tactile Image *per se*, *P*(*x, y, z*) reveals tissue or organ anatomy and elasticity distribution [5] [6] because it keeps the stress-strain relationship for deformed tissue. It is an interesting fact that the 3-D tactile image can be transformed into an elasticity image with the use a linear transformation for a region of interest. That means, in general, the spatial gradients ∂*P*(*x, y, z*)*/∂x*, ∂*P*(*x, y, z*)*/∂y* and ∂*P*(*x, y, z*)/∂*z* can be used in practical purposes for quantitative assessment of tissue elasticity because they have a validated background [1]–[6], [11]–[15] allowing quantitative comparison and analysis for different patients with anatomical variations.

The direct measurement of *in vivo* tissues presents a number of challenges. One of these challenges is that the tissue studies (such as the vaginal wall) cannot be isolated *in vivo* and the pelvic structures must be measured as a system, including the vaginal wall and underlying structures. With this in mind, it is thought that the addition of a functional muscle assessment is needed as part of the tactile image assessment to better evaluate the pelvic floor tissues and potential relative contributions of the underlying muscles. Nevertheless, the analysis of the muscle rest tone contribution into acquired parameters in Tests 1 – 3 is beyond of this study.

Currently, the most widely used assessment of pelvic organ prolapse (POP) is limited to documenting surface anatomy, such as the POP Quantification system developed by the International Continence Society [7]. More sophisticated technology, such as functional MRI and 3-D ultrasound, offer insight into anatomy with applied forces as well. And while the resulting measurements are a consequence of changes in the mechanical pelvic floor properties, they do not specifically measure the individual, mechanical properties of pelvic floor tissue [16]. These individual differences are often appreciated on a clinical exam, such as the ease that tissue descends or balloons with applied pressure, the palpable tissue properties on exam, asymmetrical defects, prior scarring, etc. The impression of the tissue quality demonstrated on an exam and the impression of what areas are affected by visual and palpated cues factor into a physician’s assessment of a patient’s condition, but are challenging to document or translate for outcomes by quantitative metrics. The terminology that describes the details of these differences is now largely descriptive and not quantified or standardized for a better comparison of baseline characteristics or a normal support conditions. A measured, reproducible soft tissue assessment by VTI may offer insight into the differences of these baseline characteristics and allow for a clearer process for determining the most effective treatment options with predictable response/outcome, with goals for optimal support as well as the maintenance of functional outcomes for underlying organ condition and function.

It is possible, that if we could make further differentiations in the biomechanical qualities of the tissues behind pelvic floor conditions, that we could offer more effective treatments. For example, better identifying compartments with “pre-prolapse” defects that may benefit from additional repair at the time of reconstructive surgery to prevent recurrent prolapse, guidance for what patients benefit from a more aggressive repair or a more limited site-specific repair, which patients may benefit from a graft material, and which patients may benefit from pelvic floor physical therapy as a conservative or peri-operative approach. Currently, the majority of these judgments rely on years of experience, resources that are available to the surgeon, surgical training backgrounds and biases and treatment preferences that the patients brings to the table. Perhaps, the VTI may offer standardized measurements to help tailor our judgments beyond the current in-office tools and to assess over time with both conservative and surgical management of pelvic floor conditions.

To date, it is challenging and important to combine functional anatomy with *in vivo* tissue properties. It would also be extremely useful to correlate mechanical properties measured by the VTI with anatomical measurements from 3-D imaging such as MRI or ultrasound. The combination of these tools, image fusions, anatomic and functional valuation may provide us the best assessment of pelvic floor conditions or a needed insight into complicated pelvic floor conditions. Another potential use for VTI data is to provide individual biomechanical data to use in the predicative modeling [17] [18] and to investigate the relationship between anatomical disruptions with the muscle function [19]–[21].

As part of the soft tissue assessment, functional imaging of the pelvic floor muscles offers a needed insight into the biomechanics of the functional pelvic floor and to help understand the relative contribution of pelvic floor muscle function to soft tissue characteristics. While urodynamics is used for the assessment of SUI conditions, there are no standardized tools to accurately acquire *in vivo* stress-strain data to evaluate the female PF for POP and SUI patients. There is a need to develop new technologies, analogous to urodynamic tests, to enable the evaluation of PF function that is quantitative, anatomically sensitive and specific. As practiced during routine pelvic floor examination for SUI, the technical need for PF diagnostics are based primarily in the sensing and measurement of the force and movement produced by musculature of the levator ani during contraction. Clearly, for a biomechanically correct delineation of PF function, it is useful to have information that is directionally sensitive and constructed to measure PF closure [22]. Earlier, the development of a vaginal probe with four force sensors for the evaluation of the dynamics of pelvic floor function was described [22]. Nevertheless, in current practice, the manual muscle testing per vagina or rectum is the technique used by most clinicians to evaluate the PF muscles. Unfortunately, due to the location of the PF muscles defining its normal function in a noninvasive way is clinically and technically challenging but possible. It is expected that by understanding the processes and, the mechanisms involved in the functioning of the PF we can better identify more sensitive clinical diagnoses and have treatment outcomes in the management of incontinence [23].

A strength of this study is that the current VTI offers an opportunity to assess the vaginal support along the entire length of the anterior, posterior and lateral walls at rest, with manually applied deflection pressures and with voluntary pelvic floor muscle contraction. This allows us a large body of measurements to evaluate individual variations in support defects as well as identify specific potential markers to measure tissue properties as they correlate to pelvic floor support. In addition, the technology gives the ability to measure pelvic floor muscle strength at specific locations along the vaginal wall and help correlate the relative contributions to measured tissue properties. These measurements may provide insight into the functional contribution or relationships between support tissue and underlying muscle support. Because VTI testing is relatively easy and inexpensive to obtain, post-treatment follow-up is obtainable to evaluate the surgical impact on functional tissue properties and pelvic floor muscles. This may provide valuable outcome measurements for evaluating our current and future treatments.

A weakness of this study is a small sample size. Further studies with larger patient population, investigating a variety of other pelvic floor conditions, and use in the evaluation of interventions including physical therapy, conservative management options and surgical correction are needed at this point to further explore diagnostic values of tactile imaging. Another weakness is the lack of data to correlate pelvic floor muscle assessment with the site of prolapse, degree of symptom severity for detected prolapse or associated urinary or fecal continence symptoms. It was thought a sub-analysis may be misleading given the limited sample size and should be reserved for future studies. There may be very important differences in functional PFM recordings between a patient with a large distention defect of the vaginal wall versus a primary apical defect, symptomatic versus asymptomatic prolapse or among patients with associated urinary or rectal complaints. For future studies, it would be important to evaluate symptom severity for pelvic floor disorders to determine whether there is a correlation between pelvic floor muscles evaluation, resting tone and associated elasticity measurements of the underlying tissue. This may help us further differentiate types of pelvic floor conditions, their underlying severity and how to tailor treatments to best care for the individual patient. An additional weakness is that the clinician obtaining the VTI measurements was not blinded to the POP-Q measurements. The procedure for VTI recording was standardized and would be difficult to bias the recording based on expectations of the measurements, however this does remain a potential bias. To diminish the potential influence of this bias, the images were evaluated and parameter values were extracted by another observer that did not have the clinical information available until the data scaling vs prolapse stage, age and parity.

## 5. Conclusion

Tactile imaging markers such as pressure, pressure gradient and dynamic pressure response during voluntary muscle contraction can be used for biomechanical characterization of female pelvic floor conditions to aid in the diagnosis and evaluation of the female pelvic floor conditions.

## Abbreviations

ANOVA: One-Way Analysis of Variance
NIA: National Institute on Aging
NIH: National Institutes of Health
HPZ: Vaginal High Pressure Zone
PF: Pelvic Floor
PFM: Pelvic Floor Muscle
POP: Pelvic Organ Prolapse
POP-Q: Pelvic Organ Prolapse Quantification System
SUI: Stress Urinary Incontinence
VTI: Vaginal Tactile Imager
